# Dual role of peripheral B cells in multiple sclerosis: emerging remote players in demyelination and novel diagnostic biomarkers

**DOI:** 10.3389/fimmu.2023.1224217

**Published:** 2023-08-10

**Authors:** Gabriel Torres Iglesias, Mireya Fernández-Fournier, MariPaz López-Molina, Dolores Piniella, Fernando Laso-García, Mari Carmen Gómez-de Frutos, Elisa Alonso-López, Lucía Botella, Beatriz Chamorro, Sara Sánchez-Velasco, Inmaculada Puertas, Antonio Tallón Barranco, Pilar Nozal, Exuperio Díez-Tejedor, María Gutiérrez-Fernández, Laura Otero-Ortega

**Affiliations:** ^1^ Neurological Sciences and Cerebrovascular Research Laboratory, Department of Neurology, Neurology and Cerebrovascular Disease Group, Neuroscience Area La Paz Hospital Institute for Health Research-IdiPAZ (La Paz University Hospital-Universidad Autónoma de Madrid), Madrid, Spain; ^2^ Immunology Department, La Paz University Hospital, IdiPAZ Health Research Institute, Madrid, Spain

**Keywords:** demyelination, serum diagnostic biomarkers, extracellular vesicles, multiple sclerosis, myelin antibodies

## Abstract

**Introduction:**

Multiple sclerosis is an inflammatory and demyelinating disease caused by a pathogenic immune response against the myelin sheath surfaces of oligodendrocytes. The demyelination has been classically associated with pathogenic B cells residing in the central nervous system that release autoreactive antibodies against myelin. The aim of the present study was to investigate whether extracellular vesicles (EVs) mediate delivery of myelin autoreactive antibodies from peripheral B cells against oligodendrocytes in multiple sclerosis (MS) and to analyze whether these EVs could mediate demyelination *in vitro*. We also studied the role of these EV-derived myelin antibodies as a diagnostic biomarker in MS.

**Methods:**

This is a prospective, observational, and single-center study that includes patients with MS and two control groups: patients with non-immune white matter lesions and healthy controls. We isolated B-cell-derived EVs from the blood and cerebrospinal fluid (CSF) and analyzed their myelin antibody content. We also studied whether antibody-loaded EVs reach oligodendrocytes in patients with MS and the effect on demyelination of B-cell-derived EVs containing antibodies *in vitro*.

**Results:**

This study enrolled 136 MS patients, 23 white matter lesions controls, and 39 healthy controls. We found autoreactive myelin antibodies in EVs that were released by peripheral B cells, but not by populations of B cells resident in CSF. We also identified a cut-off of 3.95 ng/mL of myelin basic protein autoantibodies in EVs from peripheral B cells, with 95.2% sensitivity and 88.2% specificity, which allows us to differentiate MS patients from healthy controls. EV-derived myelin antibodies were also detected in the oligodendrocytes of MS patients. Myelin antibody-loaded EVs from B cells induced myelin markers decrease of oligodendrocytes *in vitro*.

**Discussion:**

Peripheral reactive immune cells could contribute remotely to MS pathogenesis by delivering myelin antibodies to oligodendrocytes. EV-derived myelin antibodies could play a role as diagnostic biomarker in MS.

## 1 Introduction

Multiple sclerosis (MS) is a demyelinating neurological disease of the central nervous system (CNS) (1). The underlying etiology remains unclear, although the most widely accepted hypothesis is that myelin-reactive T cells trigger abnormal B cell function, including cytokine production and autoreactive antibody production against myelin antigens (2, 3). These antibody-independent and dependent mechanisms of the immune system are thought to mediate astrocyte activation, microglia reaction, and oligodendrocyte damage, leading to demyelination in the CNS of patients with MS (4).

Although multiple functions for B cells in MS have been proposed, the role of antibody-producing B cells and the identification of their MS-associated autoantibodies remain unknown (5). What is certain is that the cerebrospinal fluid (CSF) of patients with MS is characterized by deposition of clonally expanded immunoglobulins (Igs) (6), termed oligoclonal bands (OCBs), which are considered the hallmark biomarker for an MS diagnosis (7). The presence of OCBs has been classically associated with an intrathecal clonal expansion of B cells that contribute to the production of these autoreactive antibodies within the CNS (8). However, recent studies have shifted this paradigm toward the presence of clonally autoreactive B cells in the peripheral blood, suggesting peripheral B cells as another potential source of myelin autoreactive antibodies (6, 9). Whether these peripheral antibodies reach the CNS and influence MS pathogenesis remains unknown. This information is required to understand whether the immune response against myelin is initiated by peripheral immune cells or, in contrast, is originated only centrally within the CNS (6).

Extracellular vesicles (EVs) are wide-ranging groups of phospholipid bilayer nanoparticles that naturally transfer bioactive molecules (including proteins, antibodies, lipids, DNA, and RNA). EVs participate in the cross-talk of neuroimmune cell-cell communication by mediating molecule exchange (10, 11). However, whether EVs mediate antibody transfer between immune cells and neural cells is not yet known. Considering EV participation in neuroimmune communication, the present study was designed to investigate a possible dual role of antibody containing EVs in Multiple Sclerosis: I) whether EVs mediate delivery of myelin autoreactive antibodies from peripheral B cells toward oligodendrocytes resident in the CNS and the consequential demyelination; II) whether these myelin antibodies could act as a biomarker to support an optimized MS diagnosis. Deepening our knowledge of the role of peripheral B cell-derived EVs in antibody-dependent mechanisms of MS could reveal whether the peripheral arm of immunity contributes remotely to the pathogenesis of the disease.

## 2 Materials and methods

### 2.1 Study design

**Figure 1:**
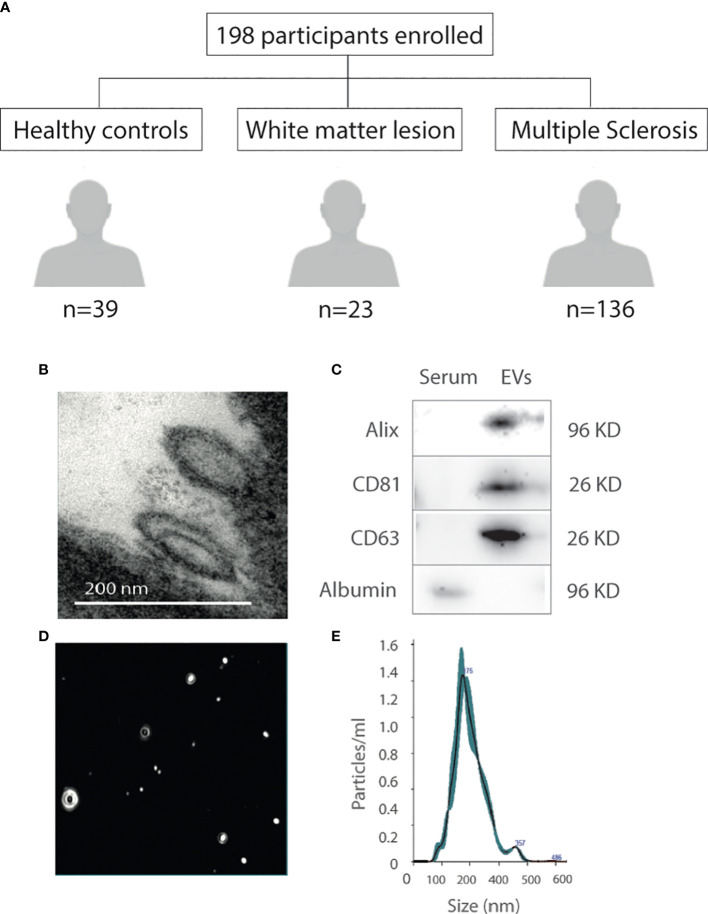
Study participants, extracellular vesicle characterization. **(A)** Flow Chart showing the study participants. **(B)** Transmission electron microscope image of EV shape and size. **(C)** EV specific tetraspanin markers (positive: Alix, CD81 and CD63; negative: Albumin) by western blot. Negative control samples are serum. **(D)** NanoSight image of circulating EVs. **(E)** Particle size and concentration analyzed by nanoparticle tracking analysis. EVs, extracellular vesicles; KDa, Kilodalton; PBS, phosphate-buffered saline.

This was a prospective, observational, single-center study that included patients (≥18 years) with relapsing-remitting MS according to the McDonald criteria (12) and who attended the MS Unit of the Neurology Department at La Paz University Hospital (Madrid, Spain) between May 2021 and October 2022. The study also included 2 control groups: I) non-immune white matter lesions: patients (≥18 years) with subcortical ischemic stroke with white matter affectation and a previous modified Rankin score ≤1; and II) healthy controls matched for age and sex (**Figure 1A**). The shared exclusion criteria were pregnancy or breastfeeding; drug or alcohol dependence; severe concomitant disease or autoimmune disease; and follow-up or participation in a clinical trial. We have included naïve patients and patients treated with interferon β, teriflunomide, dymetil fumarathe, cladribine, natalizumab and ocrelizumab. The sample is collected three months after the treatment initiation. The sample size was analyze using the G power 3.1 software, it has been estimated that for a power of 80% and a significance value of 0.05. The study was approved by the Research Ethics Committee of La Paz University Hospital (*PI-2416, PI-2562*), and all the patients signed the informed consent document. All data management was governed by the principles of Spanish Law 14/2007 of July 3 on biomedical research, ensuring the confidentiality of all personal data. The original data are available upon reasonable request.

### 2.2 Demographic and clinical data

We collected data on demographics, disease duration, activity, and the treatment received. MS disease activity was evaluated based on the occurrence of a clinical relapse or new T2 or gadolinium-enhancing lesions on magnetic resonance imaging over at least 1 year (13).

### 2.3 Extracellular vesicle isolation and characterization

Two 3-mL tubes of blood were collected from each participant. The tubes were centrifuged at 3000g for 15** **min at 4°C. To isolate circulating EVs from the blood and the CSF, we employed a commercially available EV precipitation kit (ExoQuick EV isolation kit; System Biosciences, USA), following the manufacturer’s instructions. The EVs were re-suspended in 100 µL phosphate-buffered saline (PBS). We further immunoisolated purified EVs, specifically addressing those originating from B cells and oligodendrocytes by immunoisolation with biotinylated antibodies. We incubated biotinylated anti-CD20 antibody (Merck Millipore, Germany) to obtain enriched EVs from B cells and biotinylated anti-myelin oligodendrocyte glycoprotein (MOG) antibody (Merck Millipore, Germany) to obtain those from oligodendrocytes (14), followed by incubation with Pierce Streptavidin Plus UltraLink Resin (Thermo Scientific, Inc., Waltham, MA, USA) for 30 min at 4°C. After centrifugation at 200g for 10 min at 4°C and removal of supernatant, we eluted the EVs with 0.1 M glycine-hydrochloride. After a centrifugation at 4500g for 5 min at 4°C, and the supernatants containing EVs were transferred to clean tubes. The pH was neutralized with 1 M Tris-hydrochloride.

We confirmed sequential EV immunoisolation by transmission electron microscopy (TEM), which we employed to visualize EV morphology and measure their size, as previously described (15). Moreover, we evaluated the size distribution and concentration of the purified EVs by nanoparticle tracking analysis (NTA) with the NanoSight NS500 nanoparticle analyzer (Malvern Instruments, UK). We diluted the EV samples in PBS, and captured the movement of the particles in three 60-s videos recorded at a detection threshold of 3, which we subsequently analyzed with NTA Software 2.3 (Malvern Instruments, UK). Finally, we performed western blot to analyze specific surface tetraspanin markers using the antibodies anti-Alix (1:250, Cell Signal, USA), anti-CD81 (1:250, Abcam, UK), and anti-CD63 (1:250, Abcam, UK), followed by secondary goat anti-mouse antibody (1:750, Invitrogen, USA). We employed albumin (1:1000, Abcam, UK) as a purity control. The blots were visualized with ECL Pierce chemiluminescence (Thermo Fisher Scientific, USA) in a Uvitec–Cambridge imaging system.

### 2.4 Antibody measurement

We determined the EV concentration with a bicinchoninic acid protein assay kit (Thermo Scientific, USA), following the protocol provided by the manufacturer. We disrupted the EV membrane using a Pierce radio-immunoprecipitation assay buffer (Thermo Scientific, USA). Anti-myelin basic protein (MBP), anti-MOG antibodies and anti-albumin (as negative control) were detected and quantified by sandwich enzyme-linked immunosorbent assay (ELISA) (Abbexa, UK). In short, EV contents were added to a 96-well plate coated with human MOG, MBP or albumin (Sigma-Merck Millipore, Germany). After washing, EVs were added to the plate and incubated for 60 min at 37°C. Thereafter, a working solution of detection reagent A was added to the plate and incubated for 60 min at 37°C. The wells were washed 5 times, and substrate solution was added to each well and incubated for 15 min at 37°C, in the dark. By addition of acid, the enzyme reaction was stopped and the plate was measured at 450 nm. IgG levels were calculated using a standard curve. The detection range of the ELISA kits was 3.12-200 ng/mL; the minimum detectable dose of IgG is typically less than 1.21 ng/mL. The team at IdiPAZ analyzed all antibody measurements blindly, and all assays were conducted in duplicate.

### 2.5 Culture protocol

**Figure 2:**
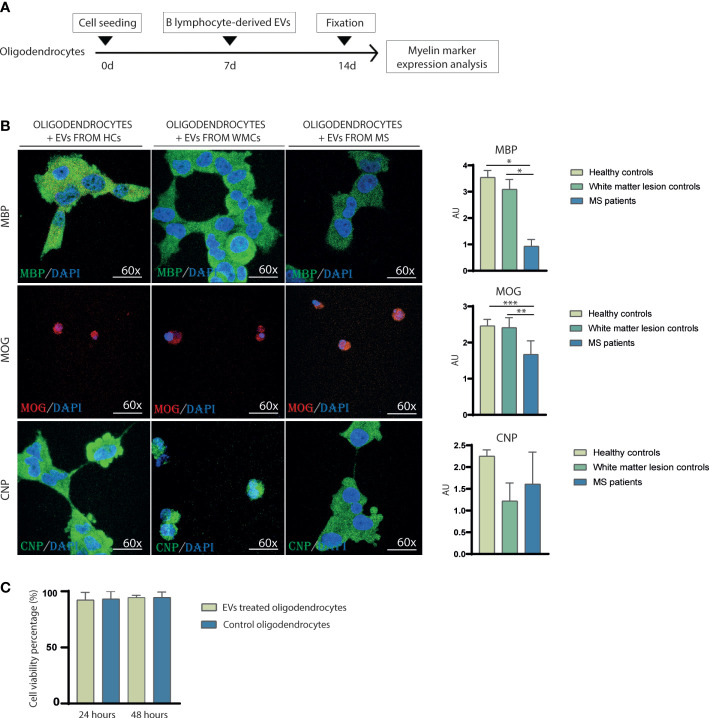
Myelin markers decrease induced by B cell-derived EVs containing myelin antibodies in oligodendrocytes *in vitro*. **(A)** Experimental protocol of *in vitro* study. Oligodendrocytes were plated and cultured using a seeding density of 1 × 10^4^ cells/cm^2^ and grown to 70% confluence. At 7 days, the cells received 100 μg of B-cell-derived EVs for 7 days. After fixing, myelin markers were analyzed. **(B)** Representative immunofluorescence images of oligodendrocytes expressing MBP, MOG, and CNP after receiving 100 µg of B-cell-derived EVs from healthy controls, white matter lesion controls, and MS cases in an *in vitro* assay. 4′,6-diamidino-2-phenylindole (DAPI) was used for nuclear staining. Quantitative analysis of MBP, MOG, and CNP marker expression by immunofluorescence (*n* = 10 assays per group). Data are mean ± SD. *p < 0.05, **p < 0.01, ***p < 0.001. **(C)** Quantitative analysis of cell viability after EV administration. Data are mean percentage ± SD. AU, arbitrary unit; CNP, 2’,3’-cyclic-nucleotide 3’-phosphodiesterase; DAPI, 4 ‘,6-diamidino-2-fenilindol; HCs, healthy controls; EVs, extracellular vesicles; MBP, myelin basic protein; MOG, myelin oligodendrocyte glycoprotein; MS, multiple sclerosis; WMC, white matter lesion controls.

We studied the ability of B cell-derived EVs containing myelin antibodies to induce demyelination *in vitro*. We acquired oligodendrocytes from Sigma-Merck Millipore and expanded them on culture flasks (Fisher Scientific), using a seeding density of 1 × 10^4^ cells/cm^2^ in Dulbecco’s Modified Eagle Medium 1x supplemented with 10% fetal bovine serum, 2 mM glutamine, 2 mM non-essential amino acids (Gibco Invitrogen, ThermoFisher, USA), and 0.01% streptomycin with 100 μg/mL penicillin G (Sigma-Merck Millipore, Germany). Cells were grown to 70% confluence and were then detached with Accutase (Sigma-Merck Millipore, Germany). All oligodendrocytes were expanded for at least 3 passages before being used for experiments. At 7 days, the cells were subjected to 100 μg of B-cell-derived EVs from patients with MS, white matter disease controls, or healthy controls. The cells were then fixed 14 days after seeding (**Figure 2A**).

### 2.6 Immunofluorescence

We analyzed myelin markers by immunofluorescence in oligodendrocytes after adding EVs. For the immunofluorescence staining, we washed the cells with PBS and fixed them with 4% paraformaldehyde for 10 min. After washing twice with PBS, we then permeabilized and blocked the cells in a solution of 1% Triton X-100 (Sigma-Merck Millipore, Germany) and 4% bovine serum albumin (BSA) in PBS for 30 min at room temperature. We incubated the cells with primary antibodies diluted in a solution of PBS containing 0.1% Triton X-100 (Sigma-Merck Millipore, Germany) and 4% BSA for 1 h. We washed the cells 3 times with PBS between primary and secondary staining, then incubated them with alexa-488 and alexa-594 secondary antibodies (1:750, Invitrogen, USA) for 30 min. The following antibodies were used for the analysis: mouse anti-MBP (1:1000; Abcam, UK), rabbit anti-MOG (1:100; Sigma-Merck Millipore, Germany), and mouse anti- 2’,3’-cyclic-nucleotide 3’-phosphodiesterase (CNP) (1:1000; Sigma-Merck Millipore, Germany). We acquired images as a maximum confocal projection, employing a Leica TCS-SPE spectral confocal microscope (Leica Microsystems, Heidelberg, Germany) with a × 60 objective lens, analyzing 6 randomly selected spot per slide. We obtained the confocal images with LAS AF software (Leica Microsystems, Heidelberg, Germany) and measured mean fluorescence intensity with the NIS-Element AR (Nikon) 4.5 Program. The team at IdiPAZ analyzed all immuofluorescence measurements blindly. We also performed a cell viability assay of the oligodendrocytes 24 and 48 hours after EVs administration using trypan blue. For cell counting, we quantified the dead cells in the four hemocytometer quadrants and we analyzed the media. This was run per triplicate and blindly. Dead cells were calculated as follows: Dead cells (%) = [(dead cells number/total cells) x 100].

### 2.7 Statistics

Data were tested for normality using the Kolmogorov–Smirnov test and the Shapiro–Wilk test. We compared the study groups’ demographics, clinical data, and antibody levels using the Kruskal–Wallis test with a *post hoc* Mann–Whitney U test for continuous variables, and Fisher’s exact test for categorical variables. We assessed the possible relationship between antibody levels and MS occurrence with logistic regression. Employing a receiver operating characteristic (ROC) analysis, we chose a cut-off point corresponding to the maximum sensitivity and specificity values to identify the antibody levels related to MS. Healthy controls and cases matched for age and sex. The team at IdiPAZ analyzed all antibody measurements and immunofluorescence blindly. No missing data in either antibody or immunofluorescence measurements. We used the Statistical Package for the Social Sciences (IBM SPSS, version 25, USA) for the analysis and GraphPad Prism 8.0.1 for the graphics. Data were mean ± SD.

## 3 Results

This study enrolled 136 patients with MS, 23 controls with white matter lesions of non-immune origin and 39 healthy controls. **Table 1** shows the demographic and clinical characteristics of the participants.

**Table 1:** Demographic and clinical data of study participants.

	HCs	WM controls	MS Patients	P-value
Demographic data				
Age (years), mean (SD)	45.26 (13.58)	64.86 (12.84)*	43.04 (9.36)	**p=0.001**
Female, N (%)	20 (51.28)	12 (47.6)	88 (64.7)	p=0.29
Clinical data				
Disease duration (months)	–		122.33 (117.47)	–
Active disease, N (%)	–		77 (56.6%)	–
Treated Patients, mean (SD)	–		54 (38.8%)	–

*Mann–Whitney U test for continuous variables and Fisher’s exact test for categorical variables were employed to determine statistically significant differences between groups. P-values <0.05 are in bold. *Age showed significant differences for WM controls and MS Patients (p=0.001). HCs, Healthy controls; MS, multiple sclerosis; SD, standard deviation; WM, white matter.

### 3.1 Purified extracellular vesicles showed typical size, morphology, and surface markers

EVs showed typical EV-like morphology and sizes of 30–300 nm by TEM (**Figure 1B**). The EVs exposed the presence of the EV-specific markers Alix, CD81 and CD63 in the EV membrane (**Figure 1C**). Albumin was used as a purity control. The population of the isolated vesicles showed the typical size distribution of EVs also analyzed by NTA (**Figures 1D, E**). These approaches allowed for robust characterization of the EV sample based on size, morphology, and tetraspanin profiles.

### 3.2 Peripheral blood does not display differences in soluble myelin antibody levels between groups

**Figure 3:**
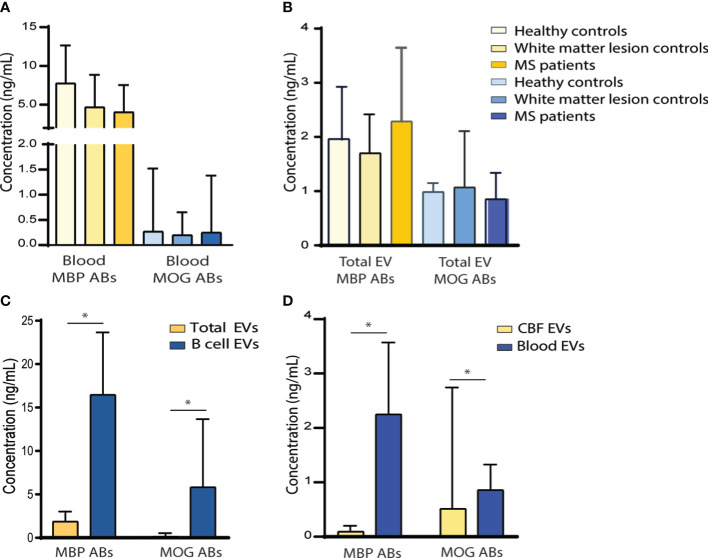
Myelin antibody content in blood and cerebrospinal fluid. **(A)** Graph showing soluble anti-MBP and anti-MOG antibodies in healthy controls (n = 39), white matter lesion controls (n = 23), and MS cases (n = 136). **(B)** Comparison of anti-MBP and anti-MOG antibody content in total circulating EVs between healthy controls, white matter lesion controls, and MS cases. **(C)** Chart comparing myelin antibody content in total circulating EVs and B-cell-derived EVs. **(D)** Myelin antibody content in B-cell-derived EVs from CSF and blood. Data are mean ± SD. *p<0.05. ABs, antibodies; CSF, cerebrospinal fluid; EVs, extracellular vesicles; MBP, myelin basic protein; MOG, myelin oligodendrocyte glycoprotein; MS, multiple sclerosis.

We first studied whether there were differences in soluble anti-MBP and anti-MOG autoantibodies in peripheral blood, between MS cases and controls. No significant differences in the levels of anti-MBP autoantibodies in the peripheral blood were found between the 39 healthy controls (7.74 ± 4.91 ng/mL), the 23 white matter lesion control group (4.69 ± 4.17 ng/mL), and the 136 patients with MS (4.05 ± 3.485 ng/mL). Similarly, no significant differences in anti-MOG autoantibody levels were found in the peripheral blood between the healthy controls (0.27 ± 1.25 ng/mL), the white matter lesion control group (0.2 ± 0.45 ng/mL), and the patients with MS (0.25 ± 1.13 ng/mL) (**Figure 3A**).

### 3.3 Myelin antibodies are not detected in total circulating extracellular vesicles from blood

We further compared the levels of anti-MBP and anti-MOG autoantibodies carried in the total circulating EVs from peripheral blood between groups. Similar levels of anti-MBP autoantibodies were found in total EVs in the 39 healthy controls (1.94 ± 0.97 ng/mL) and in the 23 white matter lesion control group (1.69 ± 0.7 ng/mL) compared with the 136 patients with MS (2.26 ± 1.32 ng/mL). No differences were found in the anti-MOG autoantibody content in total EVs among the healthy controls (0.99 ± 0.16 ng/mL), the white matter lesion control group (1.07 ± 1.01 ng/mL), and the patients with MS (0.86 ± 0.47 ng/mL) (**Figure 3B**).

### 3.4 Myelin antibodies were found in extracellular vesicles from peripheral B cells in patients with multiple sclerosis

Taking into account that antibodies are produced and secreted by B cells, we analyzed the levels of each antibody specifically in the B-cell-derived EVs and performed a comparative analysis with those antibodies found in total circulating EVs. Anti-MBP and anti-MOG antibodies were mostly found in B-cell-derived EVs (16.36 ± 7.13 ng/mL and 5.79 ± 7.77 ng/mL, respectively) compared with those found in total circulating EVs (1.85 ± 1.16 ng/mL and 0.16 ± 0.37 ng/mL, respectively) (**Figure 3C**).

### 3.5 Myelin antibodies are not detected in cerebrospinal fluid-derived extracellular vesicles

We next investigated whether B-cell-derived EV myelin antibodies were present in the CSF of 25 patients with MS and compared their levels with those found in B cell-derived EVs from blood. Significantly lower levels of anti-MBP antibodies were found in the CSF-derived EVs (0.1 ± 0.1 ng/mL) compared with those found in the blood-derived EVs (2.26 ± 1.32 ng/mL) (p=0.001). Similarly, significant differences were found in the levels of anti-MOG antibodies between CSF-derived EVs (0.52 ± 2.23 ng/mL) and blood-derived EVs (0.86 ± 0.47 ng/mL) (p=0.049) (**Figure 3D**).

### 3.6 Differential expression of myelin antibody content in B-cell-derived extracellular vesicles in patients with multiple sclerosis versus healthy controls

**Figure 4:**
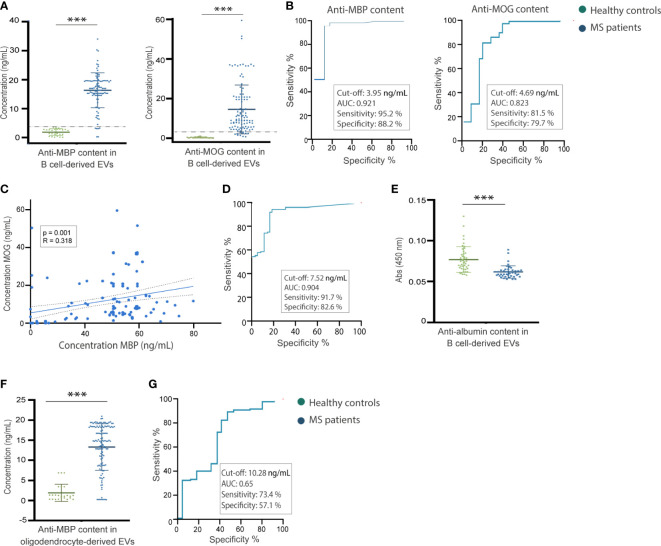
Myelin antibody content in B cell-derived EVs. **(A)** B cell-derived EV myelin antibody content in healthy controls (n = 39) and patients with MS (n = 136). **(B)** Receiver operating characteristic curves representing the cut-off point corresponding to the maximum sensitivity and specificity values in order to identify the myelin antibody levels to differentiate MS cases and healthy controls. **(C)** Correlation between EV anti-MBP and anti-MOG antibody content in MS patients. **(D)** Receiver operating characteristic curves representing the combination of anti-MBP and anti-MOG antibodies from B cell-derived EVs representing a cut-off point corresponding to the maximum sensitivity and specificity values to differentiate the MS patients from healthy controls. **(E)** Analysis of EV anti-albumin antibody content in MS patients and in healthy control. **(F)** Oligodendrocyte-derived EV myelin antibody content in healthy controls (n = 39) and patients with MS (n = 136). **(G)** Receiver operating characteristic curves representing the cut-off point corresponding to the maximum sensitivity and specificity values in order to identify the MBP antibody levels in oligodendrocyte-derived extracellular vesicles to differentiate MS cases and healthy controls.

We further analyzed the differential expression of myelin autoantibody content from B-cell-derived EVs between 136 patients with MS and 39 healthy controls. Significantly higher levels of anti-MBP and anti-MOG antibodies were found in the EVs from patients with MS (16.57 ± 5.73 ng/mL and 14.48 ± 12.37 ng/mL, respectively) compared with that of the healthy controls (1.85 ± 1.15 ng/mL and 0.16 ± 0.37 ng/mL) (p=0.001 and p=0.001, respectively) (**Figure 4A**). ROC curve analysis indicated a cut-off of 3.95 ng/mL (sensitivity: 95.2%, specificity: 88.2%) of B-cell-derived anti-MBP antibodies (area under the curve = 0.92), and a cut-off of 4.69 ng/mL (sensitivity: 81.5%, specificity: 79.7%) of B-cell-derived anti-MOG antibodies (area under the curve = 0.82) for distinguishing MS occurrence (**Figure 4B**). A positive correlation between for anti-MBP and anti-MOG antibody content of B-cell-derived EVs was found (p = 0.001 and R = 0.318) (**Figure 4C**). Combining the MBP/MOG readings, we obtained a cut-off of 7.52 ng/mL (sensitivity 91.7%, specificity 82.6% and area under the curve = 0.904) (**Figure 4D**).

In order to enlighten on whether the antibody level increase is a myelin-specific B cell response or in contrast, a general immune antibody expression, we have employed a quantification of the antibodies against the non-CNS specific antigen albumin as control. We found higher levels of antibodies against albumin in healthy controls (0.076 ± 0.015 AU) compared to MS patients (0.062 ± 0.0075 AU), p=0.001 (**Figure 4E**).

### 3.7 The oligodendrocytes of patients with multiple sclerosis also release myelin antibody-loaded vesicles

Given that myelin sheath surfaces of oligodendrocytes are the principal target of immune antibody response in MS, we evaluated whether oligodendrocytes receive these antibodies and incorporate them into their vesicles. Significantly higher levels of anti-MBP antibodies were found in oligodendrocyte-derived EVs from patients with MS (13.23 ± 5.93 ng/mL) than from healthy controls (1.52 ± 2.44 ng/mL) (p=0.001) (**Figures 4F, G**).

### 3.8 Disease modifying therapies do not modify the myelin antibody content in extracellular vesicles from patients with multiple sclerosis

**Figure 5:**
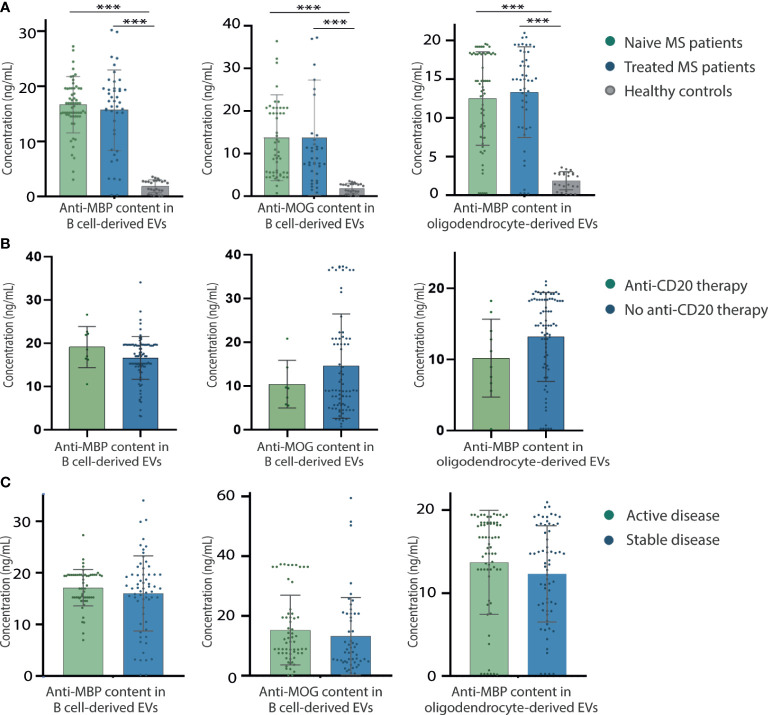
Impact of disease modifying treatments and disease activity in EVs anti-myelin antibodies content. **(A)** Myelin antibody content from B cell-derived EVs in treated patients with MS (n = 54), naïve patients (n = 82) and healthy controls (n=39). **(B)** Myelin antibody content from B cell-derived EVs in treated MS patients with anti-CD20 therapies compared with treated MS patients with different therapies. **(C)** Anti-MBP and anti-MOG levels of B cell-derived EVs, comparing patients with MS with active (n = 77) and stable disease (n = 59). Data are mean ± SD. ***p < 0.001. AUC, area under curve; EVs, extracellular vesicles; MBP, myelin basic protein; MOG, myelin oligodendrocyte glycoprotein; MS, multiple sclerosis.

We further performed a subgroup analysis to compare the anti-MBP and anti-MOG autoantibody content in EVs from 82 MS-naïve patients compared with 54 MS-treated patients. We observed similar levels of anti-MBP and anti-MOG antibody content in B cells in the MS-naïve patient group (16.41 ± 5.00 ng/mL and 13.59 ± 9.74 ng/mL, respectively) compared with the MS-treated group (15.68 ± 8.43 ng/mL and 15.64 ± 15.36 ng/mL, respectively) (p=0.62 and p=0.56, respectively). We have found higher expression in treated and naïve patients compared to healthy controls of anti-MBP (1.85 ± 1.15, p = 0.001) and anti-MOG antibodies content in EVs from B cells (0.16 ± 0.37, p = 0.001). Along these lines, similar levels of anti-MBP from oligodendrocyte-derived EVs were found in the MS-naïve patient group (12.46 ± 6.10 ng/mL) compared with the MS-treated group (13.43 ± 5.82 ng/mL, p=0.51). We have found higher expression in treated and naïve patients compared to healthy controls of anti-MBP antibodies content in EVs from oligodendrocyte (1.52 ± 2.44 ng/mL; respectively, p = 0.001) (**Figure 5A**).

We further did a sub-analysis studying anti-MBP and anti-MOG antibodies content in patients treated with anti-CD20 therapies. We did not observe significant differences between patients treated with anti-CD20 monoclonal antibody compared to other disease modifying treatments neither in anti-MBP (19.10 ± 4.74 ng/mL and 16.37 ± 5.26 ng/mL), anti-MOG (10.39 ± 5.44 ng/mL and 12.46 ± 10.57 ng/mL) from B cell-derived EV (**Figure 5B**).

### 3.9 Autoantibody levels were independent of disease activity

To analyze the correlation between anti-MBP and anti-MOG antibody content of EVs with disease activity, we performed a further subgroup analysis, further cross-defining patients with MS as active or stable. Non-significant differences were found in the quantity of anti-MBP and anti-MOG antibodies in B cells between 77 patients with MS with active disease (17.13 ± 3.52 ng/mL and 15.64 ± 11.99 ng/mL, respectively) and 59 with stable disease (16.03 ± 7.27 ng/mL and 13.19 ± 12.97 ng/mL, respectively) (p=0.29 and p=0.31, respectively). We neither found any difference between the levels of anti-MBP from oligodendrocyte-derived EVs between patients with MS with active disease (13.86 ± 6.01 ng/mL) and those with stable disease (12.47 ± 5.83 ng/mL, p=0.20) (**Figure 5C**).

### 3.10 Extracellular vesicle-derived anti-MBP and anti-MOG myelin antibodies induce myelin markers decrease

Given that anti-MBP and anti-MOG autoantibodies are thought to be implicated in demyelination, we evaluated the effects of EVs containing anti-MBP and anti-MOG autoantibodies from patients with MS on myelin markers *in vitro*. The expression of MBP protein was significantly higher in oligodendrocytes receiving B cell-derived EVs from healthy controls (3.54 ± 0.27 arbitrary units [A.U.]) and from white matter controls (3.09 ± 0.37 A.U.) than in oligodendrocytes receiving B cell-derived EVs from patients with MS (0.93 ± 0.26 A.U.) (p=0.049 and p=0.035, respectively). Moreover, expression of the MOG protein was significantly higher in oligodendrocytes receiving B cell-derived EVs from healthy controls (2.46 ± 0.18 A.U.) and from white matter controls (2.41 ± 0.28 A.U.) compared with the oligodendrocytes that received B cell-derived EVs from patients with MS (1.67 ± 0.38 A.U.) (p=0.001). The CNP signal was similar oligodendrocytes receiving B cell-derived EVs from healthy controls (2.25 ± 0.15 A.U.), white matter controls (1.22 ± 0.42 A.U.) and patients with MS (1.61 ± 0.74 A.U.) (p=0.32 and p=0.57, respectively) (**Figure 2B**). The cell viability of oligodendrocytes did not decrease 24 non 48 hours after EV administration (92.32% ± 6.71% and 94,71% ± 1,61%, respectively) when compared to oligodendrocyte receiving PBS (93,19% ± 6.75% and 94,58% ± 4,79%, respectively) (**Figure 2C**).

## 4 Discussion

Our study is the first study to identify autoreactive myelin antibodies in EVs released by peripheral B cells into the bloodstream, but not by populations of autoreactive B cells found in the CSF. Although non-significant differences were found in the levels of soluble myelin antibodies in the blood, we found higher levels of myelin autoantibodies in EVs derived from peripheral B cells in patients with MS compared with healthy controls. We identified a cut-off value of MBP autoantibodies in EVs derived from peripheral B cells which allowed us to differentiate patients with MS from healthy controls. Moreover, we detected autoantibodies in EVs from oligodendrocytes. These myelin antibody-loaded vesicles induced a decrease of myelin markers in oligodendrocytes *in vitro*. Thus, in the present study, we have delved into a possible dual role of EVs from peripheral B cells: I) as myelin-reactive antibody releasers and II) as possible diagnostic biomarkers in Multiple Sclerosis.

Myelin-specific antibodies can play an active role in MS pathogenesis (4). However, the identification of these myelin-associated autoantibodies remains to be confirmed (5). MOG and MBP are 2 of the most abundant proteins in the outer layer of the surface myelin sheath of oligodendrocytes and have been repeatedly studied as autoantigens in MS (16). A lot of studies have analyzed serum anti-MOG and anti-MBP antibodies in patients with MS, with contradictory results. Whereas typical MS cases are largely anti-MOG negative (17, 18), some studies have shown a correlation between serum anti-MOG antibodies and clinical diagnosis of the disease (19–22). Accordingly, although some studies have suggested an association between serum anti-myelin and individual risk for disease progression (19), other studies did not demonstrate a predictive value of these pathogenic antibodies for progression from clinically isolated syndrome to definite MS (23). Thus, the diagnostic value of soluble anti-MOG and anti-MBP in predicting MS remains questionable (24). The results of our study are consistent with the findings of these previous studies, given that we found similar levels of soluble anti-MOG and anti-MBP antibodies in the blood of patients with MS and healthy controls, which does not allow us to assign a diagnostic value to these soluble circulating autoantibodies. However, we found high levels of myelin autoantibodies in CD20^+^ EVs derived from peripheral B cells in patients with MS. Although the main antibody producing cells are CD19^+^ plasma cells, plasmablast and short-lived plasma cells express also CD20^+^ and we have isolated the EVs from this subpopulation in the present study (25). In addition, we have also identified a cut-off value of 3.95 ng/mL, with a high sensitivity and specificity, thereby differentiating patients with MS from healthy controls. These results suggest that those antibodies embedded in vesicles could contribute to clinical decision-making during diagnosis of disease. This biomarker would have the advantage of being minimally invasive compared with its alternative, lumbar puncture, to analyze OCBs. This is the first study to identify these antibodies in vesicles; thus, further studies will be necessary for validation of these molecules as a clinically useful diagnostic measure. In addition, in this study, we have analyzed the EV antibody content against non-MS relevant antigen in order to enlighten on whether it could be an anti-myelin specific response or, in contrast, a general antibody content increase. In this regard, we did not find an increase in general antibodies in MS patients compare to controls indicating a myelin specific immune response.

Although oligoclonal Igs in CSF has been shown to be a product of B cells resident in perivascular infiltrates and meningeal lymphoid-like follicles within the CNS (26, 27), essentially, nothing is known about how peripheral B cells participate in the establishment of these immunologically active sites in the MS brain. In our study, we found high expression of myelin autoantibodies in EVs circulating in the blood in patients with MS, whereas non-expression of these autoantibodies was found in the intrathecal CSF EVs. These results could indicate that autoreactive antibodies carried in EVs are released by reactive peripheral B cells, but not by the CSF-resident B cell population. These results are crucial to identifying novel previously unknown functions of peripheral B cells in the pathogenesis of MS, expressing vesicle-embedded autoreactive antibodies against CNS myelin. On the other hand, the isolation of CSF-derived EVs is challenging due to the small quantity and types of EVs present in this body fluid. This is a limitation of the study as this may lead to the lack of significant differences between MS patients and healthy controls. Despite the remarkable progress achieved in EV technology during the last years, some technical issues still need to be resolved regarding the isolation of EVs from cerebrospinal fluid. With the development of new advance technologies, future studies may be able to deeply explore this further.

Taking it a step further, blood EVs have the ability to cross major biological membranes, including the blood brain barrier, due to their small size and membrane composition (28). This essential characteristic provides peripheral B-cell-derived EVs the ability to reach the CNS (11). In the present study, self-reactive antibodies were also detected in EVs released from oligodendrocytes in patients with MS. Considering that the oligodendrocytes’ EV cargo originates in the endosome and its content comes from those EVs incorporated by the endocytic pathway (29), the antibodies detected in oligodendrocytes’ EVs could arise from donor cells able to incorporate myelin antibodies in their EVs and, hence, it may come from autoreactive B cells. Therefore, peripheral B cell-derived EVs could infiltrate the CNS and deliver their immunological cargo into oligodendrocytes’ endosome. However, whether these antibodies induce demyelination in oligodendrocytes from these internal structures is still unknown. In fact, the myelin antibody production could be either cause or consequence of demyelination. Interestingly, two complementary hypotheses are proposed to answer this question. The outside-in hypothesis posits a peripheral autoimmune attack against myelin, whereas inside-out implicates the immune response as a secondary autoimmune reaction against myelin debris subsequent to a primary oligodendrocyte degenerative process in the CNS (30). Which of the 2 paradigms better depicts the functions of myelin pathological antibodies in MS is debated. In our study, B-cell-derived EVs from patients with MS decreased the signal of myelin markers in oligodendrocytes *in vitro*, indicating that EV-derived myelin antibodies could be involved in the outside-in hypothesis. This result paves the way for future experiments that unveil the real contribution of these pathogenic antibodies in the patients with MS, which could potentially reveal the real mechanisms of MS pathogenesis.

After the administration of certain disease-modifying treatments (DMTs), a dose-dependent depletion of circulating B cells rapidly ensues and persists for several months, followed by a reconstitution of these cells (31). Although it has been shown that some of these DMTs could mildly affect antibody production in the CSF (4), in our study, DMTs did not affect antibody production in EVs from peripheral B cells. Despite depleting B cells, the lack of effect of DMTs on EV antibody levels could be explained by the fact that most of the treatments for MS do not primarily deplete plasmablast or plasma cells but immature B cell subsets. However, given that the B-cells derived EVs express CD20 antigen in their membrane, and this antigen is a target of anti-CD20 therapies, we did a subanalysis in order to aanalyze if these treatments affect B-cell derived EVs. In this analysis, we did not find lower expression of these antibody in their B cells-derived EVs, demonstrating that this type of disease modifying treatment do not affect antibody-producing vesicles. The blood collection was only three months after treatment initiation, future studies may elongate this time interval to verify whether EV anti-CD20 antibodies could be still detected. However, these are preliminary findings and future studies may clarify the impact of treatments on the MS immunophenotype of EVs to improve B-cell control and direct us to personalized treatment.

In conclusion, this study identified autoreactive myelin antibodies in EVs released by peripheral B cells that cause decreased the signal of myelin markers *in vitro*, indicating that peripheral reactive immune cells might contribute by contact-independent mechanisms to the pathogenesis of MS. These results trigger a call for a reconsideration of peripheral immune response contributions to MS pathogenesis.

These myelin autoantibodies in peripheral B cell-derived EVs could act as a minimally invasive diagnostic biomarker, helping to address the need for accurate biomarkers for clinical support in MS and to clarify the underlying cause of MS in the coming years.

## Data availability statement

The raw data supporting the conclusions of this article will be made available by the authors, without undue reservation.

## Ethics statement

The studies involving human participants were reviewed and approved by The study was approved by the Research Ethics Committee of La Paz University Hospital (PI-2416, PI-2562). The patients/participants provided their written informed consent to participate in this study.

## Author contributions

GTI, acquisition and analysis of data. MF-F, acquisition and analysis of data. DP, acquisition and analysis of data. FL-G, acquisition and analysis of data. MG-dF, acquisition and analysis of data. ML-M, acquisition and analysis of data. EA-L, acquisition and analysis of data. LB, acquisition and analysis of data. BC, acquisition and analysis of data. SS-V, acquisition and analysis of data. IP, acquisition and analysis of data. ATB, acquisition and analysis of data. ED-T, conception and design of the study, drafting a significant portion of the manuscript or figures. MG-F, conception and design of the study, acquisition and analysis of data, drafting a significant portion of the manuscript or figures. LO-O, conception and design of the study, acquisition and analysis of data, drafting a significant portion of the manuscript or figures.
