# Supplementary figures and images for: Dual role of peripheral B cells in multiple sclerosis: emerging remote players in demyelination and novel diagnostic biomarkers
Source: Front Immunol. 2023 Aug 10;14:1224217. doi: 10.3389/fimmu.2023.1224217 (PMC10449256; doi:10.3389/fimmu.2023.1224217)

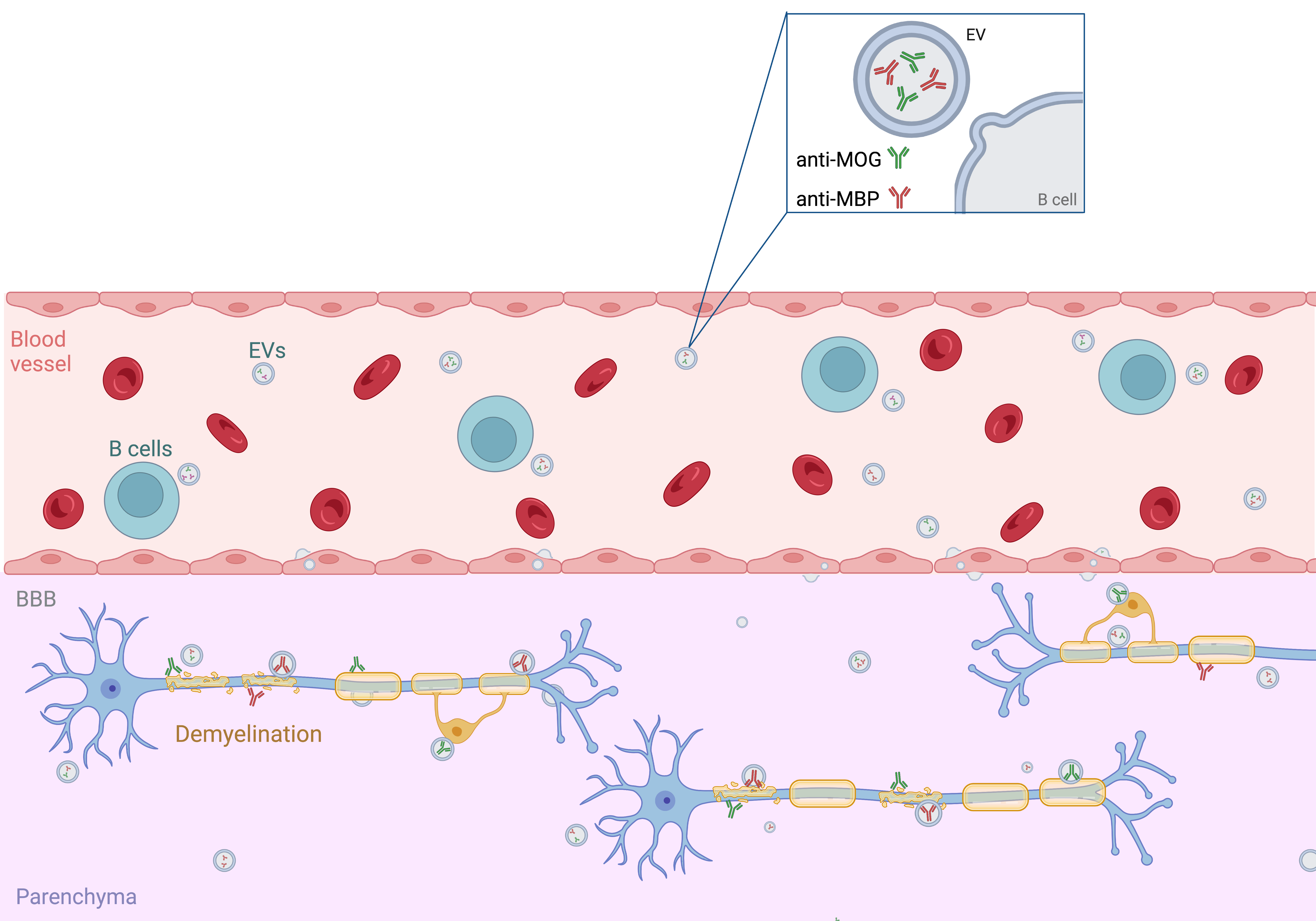

Supplement: Supplementary file 1 [file Image_1.png]
